# Cooperative supramolecular polymerization of an amine-substituted naphthalene-diimide and its impact on excited state photophysical properties†
†Electronic supplementary information (ESI) available: Synthesis, experimental procedure and supporting data. See DOI: 10.1039/c5sc03462k


**DOI:** 10.1039/c5sc03462k

**Published:** 2015-10-30

**Authors:** Haridas Kar, Dominik W. Gehrig, Naveen Kumar Allampally, Gustavo Fernández, Frédéric Laquai, Suhrit Ghosh

**Affiliations:** a Polymer Science Unit , Indian Association for the Cultivation of Science , 2A & 2B Raja S. C. Mullick Road , Kolkata-700032 , India . Email: psusg2@iacs.res.in; b Max Planck Research Group for Organic Optoelectronics , Max Planck Institute for Polymer Research , Ackermannweg 10 , D-55128 Mainz , Germany; c Institut für Organische Chemie and Center for Nanosystems Chemistry , Universität Würzburg , Am Hubland , 97074 Würzburg , Germany

## Abstract

A donor–acceptor–donor (D–A–D) type naphthalene-diimide (NDI-H) chromophore exhibits highly cooperative J-aggregation leading to nanotubular self-assembly and gelation in *n*-decane, as demonstrated by UV/Vis, FT-IR, photoluminescence and microscopy studies. Analysis of temperature-dependent UV/Vis spectra using the nucleation–elongation model and FT-IR data reveals the molecular origin of the cooperative nature of the self-assembly. The supramolecular polymerization is initiated by H-bonding up to a degree of polymerization ∼20–25, which in a subsequent elongation step promotes J-aggregation in orthogonal direction leading to possibly a sheet-like structure that eventually produces nanotubes. Time-resolved fluorescence and absorption measurements demonstrate that such a tubular assembly enables very effective delocalization of excited states resulting in a remarkably prolonged excited state lifetime.

## Abstract 2

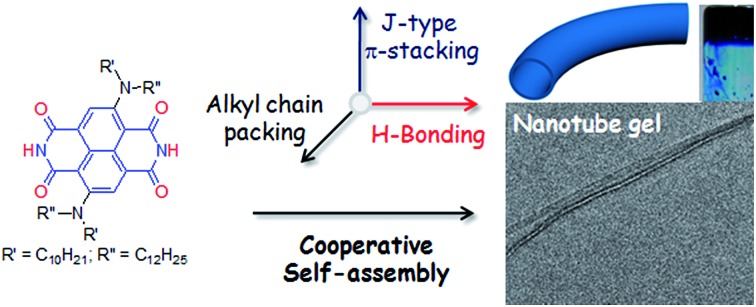
A donor–acceptor–donor (D–A–D) type naphthalene-diimide (NDI-H) chromophore exhibits highly cooperative J-aggregation leading to nanotubular self-assembly and gelation in *n*-decane.

## Introduction

**Scheme 1:**
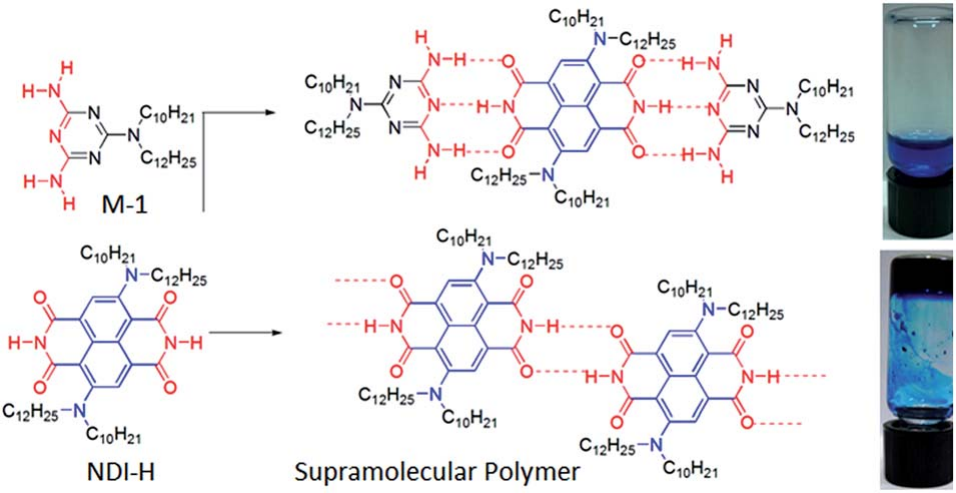
H-bond driven supramolecular polymerization and gelation (right, bottom) of NDI-H in *n*-decane (*c* = 4.0 mM). The addition of a DAD H-bonding competitor (M-1 (2.0 equiv.)), prevents the gelation (right, top).

Self-assembled π-conjugated small molecules have found widespread interest owing to their diverse photophysical and transport properties.^1^ Supramolecular polymerization^2^ of such π-conjugated monomers^3^ could be promising to make them more relevant in miniaturized organic device applications as on the one hand it offers the structural precision of oligomers, while at the same time includes features of polymers such as phase separation, film formation and others. Core-substituted naphthalene-diimides (cNDIs),^4^ owing to their wide range of light absorption, fluorescence and electrochemical properties, have been extensively studied^5^ in the context of organic solar cells, field effect transistors, photo-induced electron transport, bio-sensing, anion binding, transport and catalysis. However, there is no report on supramolecular polymerization of any cNDI and in fact only a few reports describe their non-covalent assembly.^6^ Herein we reveal for the first time H-bonding-initiated supramolecular polymerization of a diamino-substituted cNDI^4^ (NDI-H, Scheme 1, for synthesis see ESI†), the thermodynamic aspects, molecular origin of its cooperative nanotubular self-assembly and pronounced effects on the dynamics of the charge-separated state.

## Results and discussion

NDI-H was synthesized (Scheme S1†) from commercially available naphthalene-tetracarboxylic-acid-dianhydride and isolated in pure form with an overall yield of 19%. It contains two electron donating amine functionalities in the electron deficient ring, free NH groups for extended H-bonding driven supramolecular polymerization and the branched alkyl chains to provide solubility in less polarizable organic solvents where H-bonding can be prominent. Its absorption spectrum in THF (Fig. 2a) exhibits sharp absorption bands with vibronic features in the region of 300–450 nm owing to π–π* transitions along with an intense band around 500–600 nm originating from the intra-molecular charge transfer (ICT) according to literature precedence. This is more evident from the redox potentials of NDI-H as shown by cyclic voltammetry (Fig. S1†). It reveals two reversible reductions at –0.85 V and –1.06 V *vs.* Fc/Fc^+^ due to the formation of radical anions and dianions, respectively, suggesting that amine substitution slightly destabilizes the radical anions as the values are shifted towards a more negative region compared to the ring-unsubstituted NDIs. However, interestingly, now radical cation formation is also apparent as evidenced by two reversible oxidation waves at +1.02 V and +1.28 V. This unique feature of cNDIs makes them comparable to chlorophylls. Based on these data, the HOMO and LUMO energy levels can be estimated to be –5.43 eV (reference: –5.1 eV for Fc) and –3.56 eV, respectively, suggesting increases in both energy levels compared to unsubstituted NDI as also reported earlier.^4^

The supramolecular polymerization of NDI-H (Scheme 1) was examined in a few organic solvents (Table S1†) and gelation was observed in *n*-decane (Scheme 1). The lack of gelation in the presence of M-1 (Scheme 1), having a DAD type complementary H-bonding motif, suggests that supramolecular polymerization by extended H-bonded chain formation among the imide groups of the NDI-H is crucial for the observed gelation to occur.

**Fig. 1:**
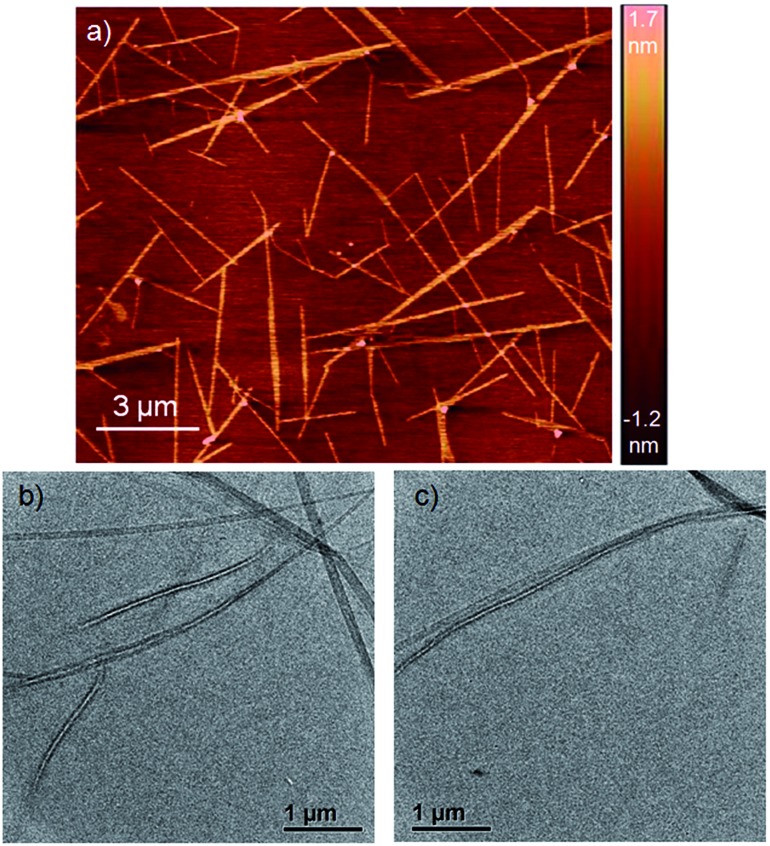
(a) AFM images of a diluted NDI-H gel on a mica surface. (b and c) HRTEM images of NDI-H nanotubes in *n*-decane on a carbon-coated copper grid.

**Fig. 2:**
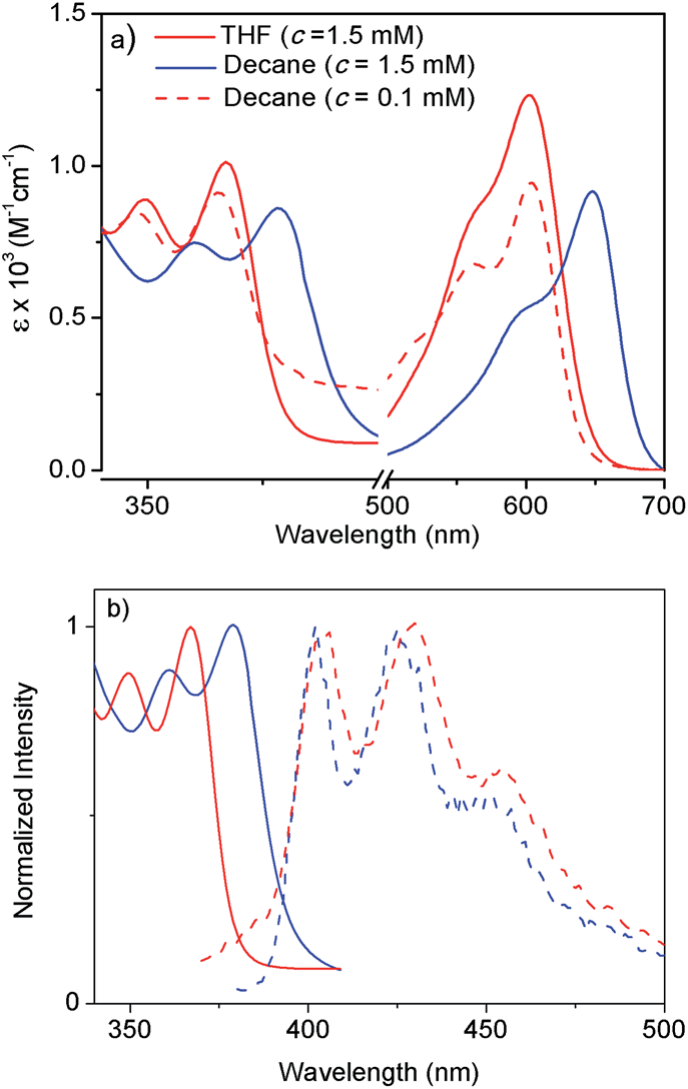
(a) Solvent- and concentration-dependent UV/Vis spectra (intensity normalized with concentration) of NDI-H (*l* = 0.1 cm, *T* = 298 K). (b) Intensity normalized absorption and emission (*λ*_ex_ = 350 nm) spectra of NDI-H in THF (red) and *n*-decane (blue). Solid line and dotted line denote absorption and emission respectively.

Atomic force microscopy (AFM) images (Fig. 1a and S2†) show one-dimensional few-micrometer long structures^7^ with widths in the range of 100–150 nm. High resolution transmission electron microscopy (HRTEM) images of a diluted gel reveal nanotubular structures^8^ (Fig. 1b and c and S3†) with diameters and lengths of 81 ± 6 nm and >5.0 μm respectively, and thus complement the AFM images. UV/Vis absorption spectra of NDI-H show pronounced solvent effects. While in THF it exhibits monomeric features, in *n*-decane a pronounced bathochromic shift of *ca.* 12–13 nm is noticed (Fig. 2a) for the π–π* bands indicating J-aggregation.^9^ The ICT-band shows an even more pronounced bathochromic shift of ∼50 nm indicating a reduction of the HOMO–LUMO energy gap due to effective delocalization of the CT-state in J-aggregation. Interestingly, the spectrum (Fig. 2a) of NDI-H in decane below a critical aggregation concentration (CAC) is similar to that of the polymer in THF, indicating that the observed changes are indeed related to aggregation and not mere solvatochromism.

UV/Vis studies in a few other nonpolar organic solvents (Fig. S4a†) reveal J-aggregation only in aliphatic hydrocarbons. In highly polar media (1 : 1 THF/MeOH) the CT-band exhibits a bathochromic shift of ∼15 nm (Fig. S4b†) compared to aliphatic solvents due to the solvatochromic effect. Concentration-dependent absorption studies (Fig. S5†) indicate a CAC of ∼0.2 mM. Sharp emission bands are observed in *n*-decane (Fig. 2b) in conjunction with a very small Stokes shift (∼24 nm). The emission exhibits a mirror-image-like symmetry with the absorption confirming J-aggregation.

**Fig. 3:**
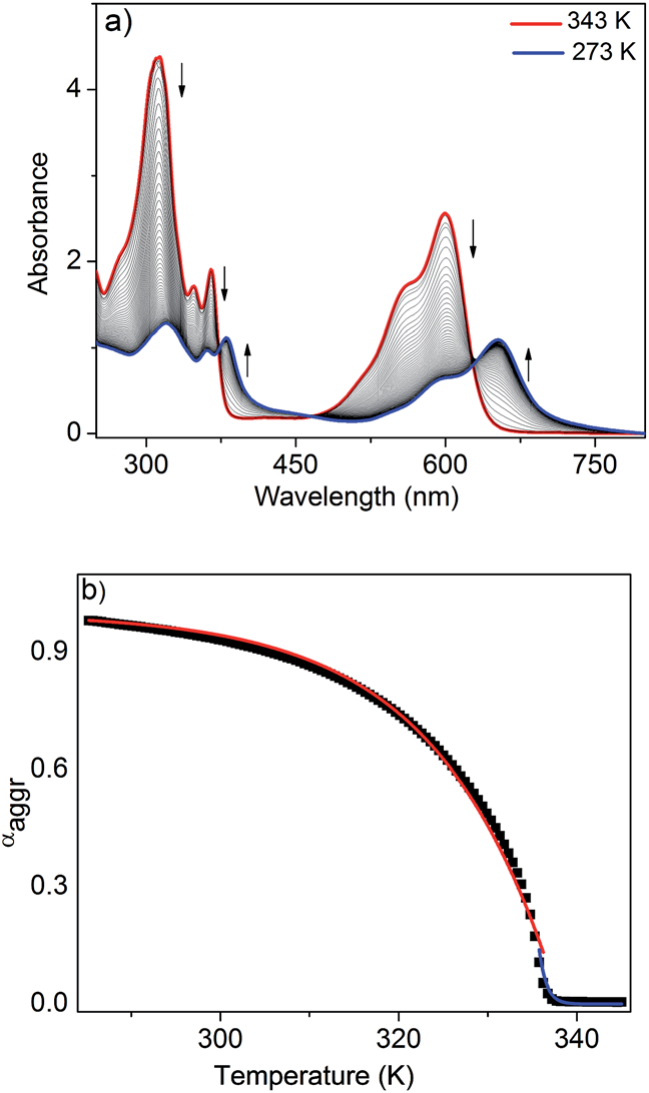
(a) Temperature-dependent UV/Vis experiments of NDI-H (1.75 mM, decane). Arrows indicate the spectral changes upon decreasing the temperature. (b) *α*_agg_ at 599 nm against temperature and fits to the cooperative nucleation–elongation model. Red and blue lines indicate the fits to the elongation and nucleation regimes, respectively.

To further analyze the J-aggregation, temperature-dependent UV/Vis studies were carried out in *n*-decane at six different concentrations (0.6–1.75 mM). Fig. 3a shows the temperature-dependent UV/Vis spectra of NDI-H (*c* = 1.75 mM, cooling rate: 1 K min^–1^). At 343 K, NDI-H exhibits the characteristic absorption features of the monomeric species. Upon cooling, depletion of the absorption maxima at 312, 348, 365 and 599 nm occurs at the expense of new red shifted transitions at 318, 361, 381 and 652 nm that can be assigned to J-aggregation. The appearance of isosbestic points at 373, 468 and 627 nm is indicative of a thermodynamic equilibrium between monomeric and self-assembled species. When the fraction of aggregated species (*α*_agg_) at 599 nm (see ESI† for details) for all six concentrations were plotted against temperature, sharp non-sigmoidal curves were obtained (Fig. 3b and S6†) indicating cooperative self-assembly.^10^

To analyze the data, we have made use of both the nucleation–elongation model developed by ten Eikelder, Markvoort and Meijer (which assumes an initial dimerization process followed by a more favorable elongation step)^11^ as well as the model for thermally-activated equilibrium polymers described by van der Schoot.^12^ The latter distinguishes between a nucleation regime (characterized by a dimensionless equilibrium constant *K*_a_ that provides a measure of the degree of cooperativity) in which a monomeric activation step occurs, followed by a subsequent elongation step at lower temperatures (described by the elongation constant *K*_e_). Both regimes are separated by the elongation temperature *T*_e_. Although both models yielded satisfactory fits (see ESI†), we found that the van der Schoot model describes the experimental data more accurately in the high-temperature nucleation regime at all six concentrations (Fig. 3b, S6 and S7†). The remarkably low values of *K*_a_ (6 × 10^–5^ to 1 × 10^–4^) (Table 1) reveal that the self-assembly of NDI-H occurs in a highly cooperative manner. The *T*_e_ ranges from 321 to 336 K, whereas the binding constant associated to the elongation step (*K*_e_) was calculated to be in the range of 0.57 to 1.7 × 10^3^ M^–1^ (Table 1). Interestingly, the model also yields an average degree of polymerization (*N*) of 20–25 at *T*_e_.

**Table 1:** Thermodynamic parameters of the cooperative self assembly of NDI-H

Conc./M	*N*	Δ*H*°e/kJ mol^–1^	*K*_e_/M^–1^	*T*_e_/K	*K*_a_
0.6 × 10^–3^	22	–79.1	1.66 × 10^3^	321.9	9.3 × 10^–5^
0.8 × 10^–3^	21	–73.7	1.25 × 10^3^	326.5	1.1 × 10^–4^
1.0 × 10^–3^	26	–70.5	0.89 × 10^3^	328.1	5.6 × 10^–5^
1.25 × 10^–3^	24	–70.6	0.79 × 10^3^	329.3	6.9 × 10^–5^
1.50 × 10^–3^	25	–71.3	0.67 × 10^3^	336.0	5.9 × 10^–5^
1.75 × 10^–3^	25	–79.9	0.57 × 10^3^	336.7	6.1 × 10^–5^

**Fig. 4:**
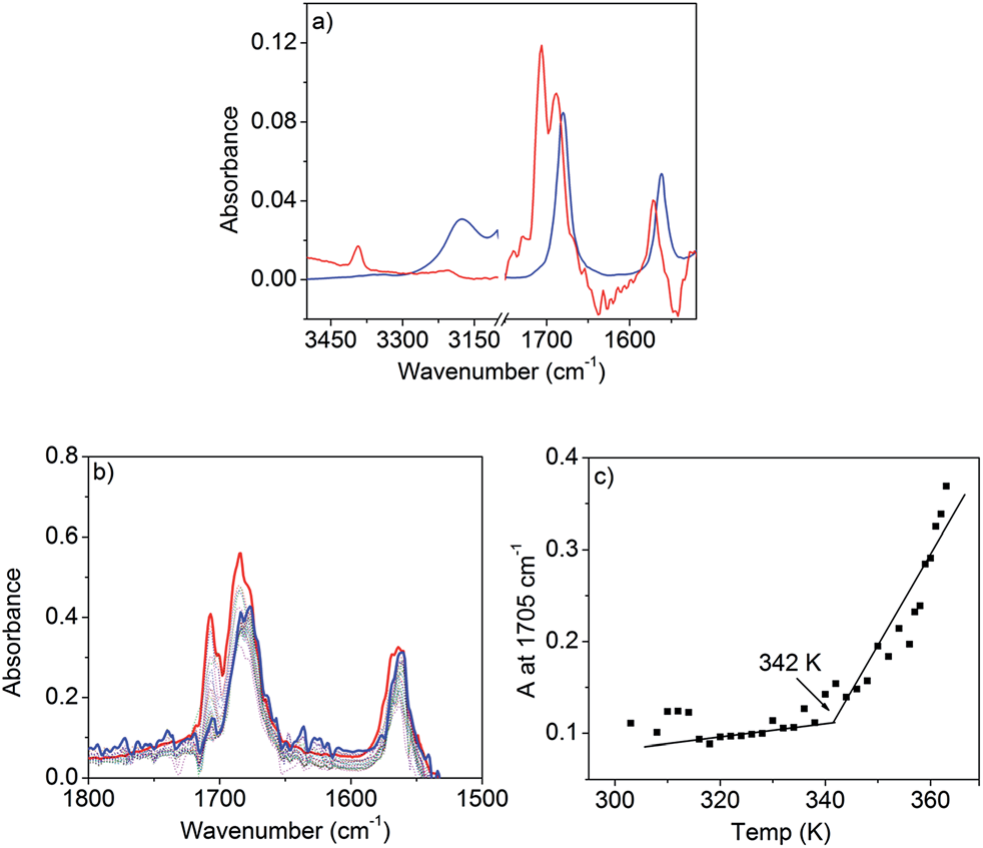
(a) FT-IR spectra of NDI-H in *n*-decane at 1.5 mM (gel, blue line) and 0.1 mM (sol, red line). (b) Variable temperature FT-IR for NDI-H (*c* = 1.5 mM) in *n*-decane. (c) Plot of absorbance at 1705 cm^–1^*vs. T*.

FT-IR studies in *n*-decane (*c* = 0.1 mM, below CAC) show sharp peaks (Fig. 4a) at 3390 and 1571 cm^–1^ for the stretching and bending vibrations of the N–H, respectively. Two other sharp bands at 1705 and 1688 cm^–1^ can be assigned to the asymmetric and symmetric stretching vibrations of the C=O of the imide group.

Above CAC (1.5 mM), the NH stretching band appears at a lower frequency (3170 cm^–1^) and the NH bending peak shifts to 1560 cm^–1^ supporting H-bond formation. Similarly, the C=O bands shift towards a lower frequency and appear as a single peak at 1678 cm^–1^ suggesting that asymmetric stretching becomes IR inactive in the aggregated state. The spectrum at lower concentrations appears similar (Fig. S8†) to that in THF or CHCl_3_ confirming that the spectral shift above CAC is due to H-bonding. Variable temperature (363 K to 303 K) FT-IR studies (Fig. 4b) in *n*-decane (*c* = 1.5 mM) show a gradual transformation of the monomeric spectrum to an aggregated spectrum as the two sharp peaks (C=O stretching) converge to one broad peak at lower temperatures. A plot of the peak intensity at 1705 cm^–1^ with temperature (Fig. 4c) shows an inflection point at ∼342 K indicating that the H-bonding is almost saturated. UV/Vis studies show (Fig. 3b) the onset of a π–π interaction at around the same temperature and thus suggest the π-stacking to be a consequence of H-bonding, therefore explaining the origin of the cooperativity.

**Fig. 5:**
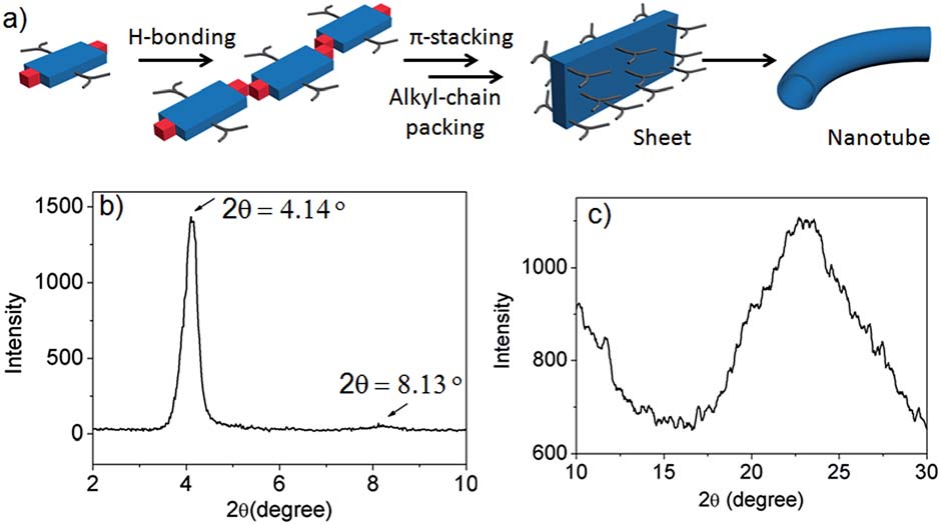
(a) Proposed model for self-assembly of NDI-H into nanotubes by H-bonding driven 1D assembly possibly followed by sheet formation by π-stacking and alkyl chain packing in orthogonal directions. (b and c) XRD pattern of dried NDI-H gel.

Thus we propose (Fig. 5a) that NDI-H initially undergoes a linear oligomerization by H-bonding.^3^ When the length of the oligomer becomes sufficiently large (*N* at *T*_e_ ∼ 20–25, as revealed by the nucleation–elongation model), J-aggregation and alkyl-chain packing come to the fore, leading to the formation of a 2D sheet that eventually bends to generate nanotubes. However, the proposed structures for the intermediates of the self-assembly pathway could not be fully elucidated by experimental data. The small angle powder X-ray diffraction (XRD) pattern shows (Fig. 5b) a sharp reflection (100) at 2*θ* = 4.14°, corresponding to *d* = 20.8 Å, closely matching the estimated width of NDI-H (Fig. S9†) across the longer diagonal axis. The presence of a peak at a distance of 10.5 Å (*d*/2) supports the proposed lamellar packing. The wide angle X-ray diffraction (XRD) pattern shows (Fig. 5c) a broad peak in the region of 2*θ* = 17–29° (corresponding to *d* = 5.10–3.01 Å) that may arise due to alkyl chain packing and π–π stacking. Interestingly HRTEM images of a sample prepared from a diluted solution of NDI-H in decane show (Fig. S10†) a sheet-like morphology supporting the proposed model.

**Fig. 6:**
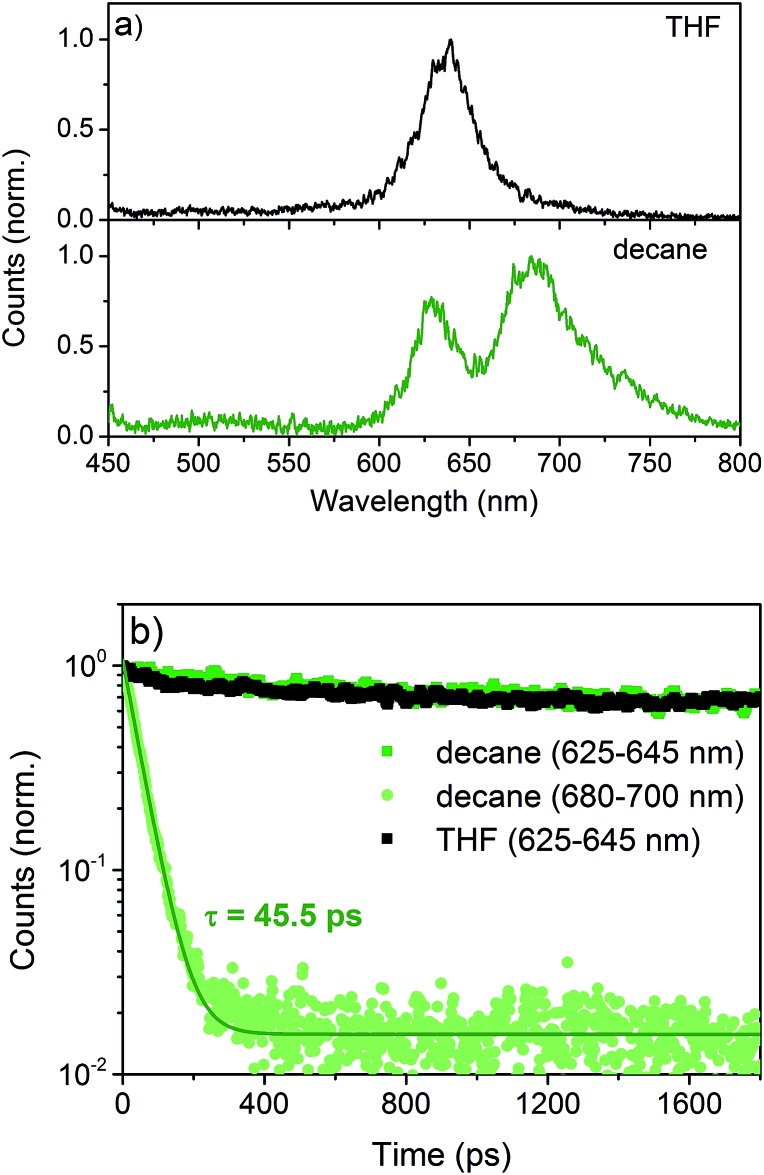
(a) Emission spectra of NDI-H in THF and decane. (b) PL decay followed at the monomeric emission from 625–645 nm in THF (black filled symbols) and decane (green square symbols) and from 680–700 nm in decane (green circular symbols) together with a monoexponential fit (green solid line).

Time-resolved photoluminescence (TR-PL) and transient absorption (TA) spectroscopy were performed to investigate the effects of aggregation on the excited state dynamics.^6^ NDI-H exhibits an emission which peaks at 630 nm both in THF and *n*-decane (Fig. 6a) with very similar lifetimes. This is assigned to the radiative decay of singlet excited states localized on NDI monomers, as they are the only emissive species in THF. An additional emission at 680 nm emerges in *n*-decane showing an inverse decay rate of 45.5 ps (Fig. 6b). This is very fast compared to the monomeric emission, which shows only a negligible decay on the timescale of this experiment. This fast decay is assigned to the delocalization of the excitation energy over a large number of J-aggregated NDI-H molecules^9^ that leads to the population of a non-radiative dark state.

**Fig. 7:**
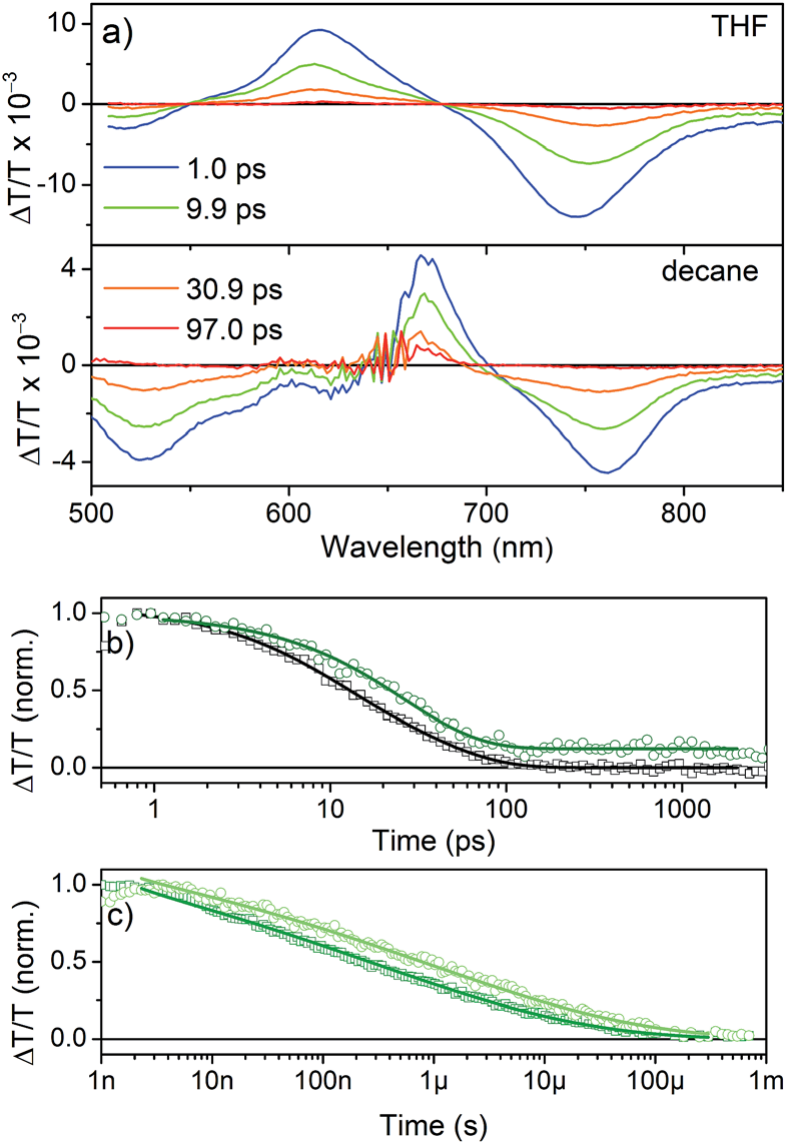
(a) Transient absorption spectra after excitation at 650 nm in THF and *n*-decane at different time delays. (b) ps–ns kinetics tracked at the respective maxima (open symbols) in THF (black) and *n*-decane (green) and (bi)exponential fits (solid lines). (c) ns–μs kinetics (in decane) extracted at 670–690 nm (dark green open symbols) and 710–730 nm (light green open symbols) and fits applying a stretched exponential decay function (solid lines).

TA spectra (Fig. 7a) show a positive feature that peaks at 615 nm in THF and 667 nm in *n*-decane, respectively. The spectral range coincides with the ground state absorption (Fig. 2a) and thus is assigned to the ground-state bleach (GSB). The shift of the GSB is consistent with the shift of the ground-state absorption spectrum from 600 nm to 652 nm upon aggregation. At shorter and longer wavelengths negative signals are observed, which are assigned to the photo-induced absorption of excited singlet states, as they appear immediately after excitation. The transient signal measured in THF (Fig. 7b) decays within ∼200 ps with inverse decay rates of 8.3 ps and 34 ps as obtained from a biexponential fit to the data. On the other hand, aggregated NDI-H in *n*-decane shows a decay with an inverse decay rate of 26 ps with an additional offset accounting for >10% signal intensity remaining even beyond 3 ns.

**Fig. 8:**
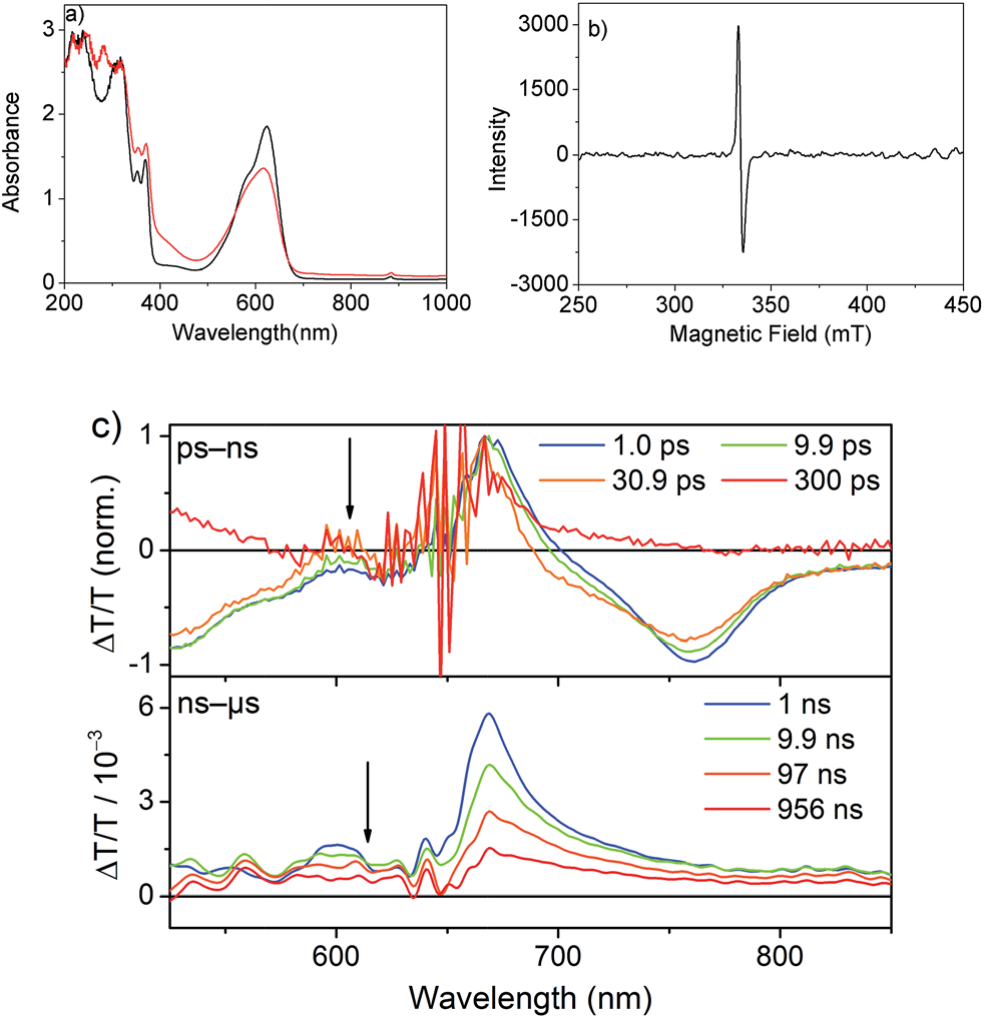
(a) UV/Vis spectra of NDI-H before (black) and after reducing it with 2.0 equiv. of cobaltocene (red) in DCM. *T* = –10 °C, *c*(NDI-H) = 0.25 mM, *l* = 1.0 cm. (b) EPR spectra (microwave frequency: 9.13 GHz, modulation width: 1 mT, microwave power: 0.998 mW, time constant: 0.03 s) of the reduced species in DCM at 77 K. (c) Normalized ps–ns transient spectra (upper panel) showing the spectral evolution of the signal and ns–μs transient spectra (lower panel).

The spectrum after a few hundred picoseconds has a different shape (upper panel, Fig. 8c). The spectral shape observed at long delays, that is, in the ns measurement, is preserved on the ns–μs timescale (Fig. 8c, lower panel). The decay proceeds on the ns–μs timescale and is very slow extending beyond 100 μs (Fig. 7c). Interestingly, on this long time scale no PIA can be observed and the positive feature extends to wavelengths longer than 800 nm (Fig. 8c) and thus we assign it to delocalized charged states. The positive feature can be assigned to stimulated emission from aggregates, as this wavelength range is consistent with the emission observed in TR-PL experiments (Fig. 6a). As this emission is very short-lived in PL experiments (inverse decay rate of 45.5 ps) we exclude the possibility of it originating from triplet states. Previous literature reports NDI anion-induced absorption at 610 nm.^13^ To further strengthen our assignment, we reduced NDI-H chemically with cobaltocene^14^ and obtained the NDI-H anion spectrum which peaks at 609 nm (Fig. 8a). In the EPR spectrum, the reduced species shows the presence of a radical (Fig. 8b). At this wavelength our TS spectra show a feature on the ps–ns time scale (Fig. 7a, lower panel and Fig. 8c, upper panel) and a dip in the positive spectrum on the ns–μs timescale (Fig. 8c lower panel indicated by black arrow) which is indicative of the presence of negatively charged NDI radical species.^15^

Therefore we can assign the origin of the long lived signal to the delocalization of the excited state over several NDI-H molecules in J-aggregates. As the number of NDI-H molecules stacked in aggregates is not the same for all aggregates and as an ensemble of aggregates is probed by TA spectroscopy, the decay is best described by a stretched exponential decay function. Such a function can be used to describe decays in systems that exhibit a distribution of lifetimes, which is very likely in this system.

## Conclusion

Overall we have elucidated the mechanistic details and molecular origin of a highly cooperative self-assembly pathway involving H-bonding, π-stacking and alkyl chain packing that leads to nanotube formation from a simple diamine-substituted NDI-dye. This is unprecedented in the literature of supramolecular assembly of any cNDI.^4^ J-aggregated dye molecules encapsulated in the multilayer walls of the tubes facilitate very effective delocalization of the excited states leading to remarkably prolonged excited state lifetimes, which is highly desirable in emerging areas including photocatalysis^16^,^17^ and light harvesting. Although cNDIs originate from the NDI skeleton, they have distinctly different photophysical and redox properties and thus offer a much broader scope as emerging organic materials. Considering the possibility of accessing numerous NDI-H analogues by different ring substitution reactions and their diverse photophysics, this particular design for cooperative supramolecular polymerization will significantly enhance the impact of cNDIs in organic electronics.

## Supplementary Material

Supplementary information
