# Supplementary material for: Cooperative supramolecular polymerization of an amine-substituted naphthalene-diimide and its impact on excited state photophysical properties
Source: Chem Sci. 2015 Oct 30;7(2):1115–20. doi: 10.1039/c5sc03462k (PMC5975828; doi:10.1039/c5sc03462k)
Supplement: Supplementary file 1 [file SC-007-C5SC03462K-s001.pdf]

Electronic Supplementary Material (ESI) for Chemical Science.
This journal is © The Royal Society of Chemistry 2015

Supporting Information for

Cooperative supramolecular polymerization of an amine-substituted naphthalenediimide and its impact on excited state photophysical properties

Haridas Kar,[a] Dominik W. Gehrig,[b] Naveen Kumar Allampally,[c] Gustavo Fernández,*[c] Frédéric Laquai*[b] and Suhrit Ghosh*[a]

[a] Polymer Science Unit, Indian Association for the Cultivation of Science, 2A & 2B Raja S. C. Mullick Road, Kolkata, 700032 India; [b] Max Planck Research Group for Organic Optoelectronics , Max Planck Institute for Polymer Research, Ackermannweg 10, D-55128 Mainz, Germany; [c] Institut für Organische Chemie and Center for Nanosystems Chemistry, Universität Würzburg, Am Hubland, 97074 Würzburg, Germany

Materials and methods: Napthalene anhydride, dodecyl amine, 1-bromodecane and 6-choloro-1,3,5 triazine-2,6 diamine were purchased from the Sigma Aldrich Chemical Co. and used without further purification. Solvents were purchased from Merck-India and purified by reported protocol.[1] For physical studies spectroscopic graded solvents have been used.^{1}H NMR and ^{13}C NMR experiments were performed on a Bruker DPX-300 MHz and 500 MHz NMR machine and all the data were calibrated against TMS. UV/ Vis absorption experiments were performed on a Perkin-Elmer Lambda 25 spectrometer. Mass spectrometric data were acquired by an electron spray ionization (ESI) technique on a Q-tof-micro quadruple mass spectrometer (Micro mass). MALDI-TOF MS data were recorded using Bruker Daltonics flex Analyser. Transmission electron microscopy (TEM) images were captured by using a JEOL-2010EX machine operating at an accelerating voltage of 200 kV. Atomic force microscopy (AFM) was performed on Innova instrument from Bruker in tapping mode. FT-IR spectra were recorded in a Perkin Elmer Spectrum 100FT-IR spectrometer. Transient absorption spectroscopic measurements were performed with a home-built pump-probe setup. EPR experiment was performed in Jeol (JES FA 200) spectrophotometer. Chemical reduction was monitored in Agilent 8453 diode arrays at -10˚C.

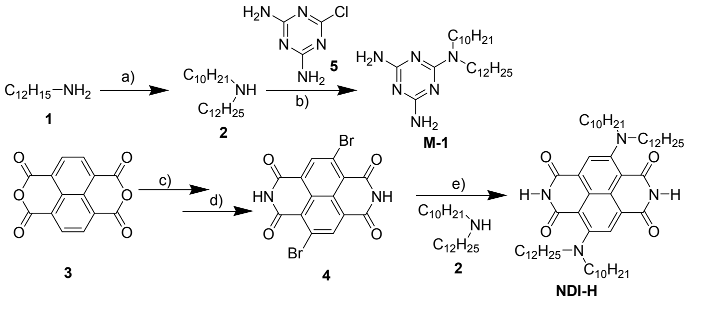

Reagents and conditions : a)1-bromodecane, methanol, 80^{0}C, 24h, 43% ; b) $NaHCO_3$, DMF, 140^{0}C, 16h, 80% ; c) 1,3 dibromo-5,5-dimetylhydantoin, H_2SO_4, 50^{0}C, 18h, 72% ; d) NH_4OAC, acetic acid,100^{0}C, 3h, 50% ; e) *N*-decyldodecan-1-amine, dry DMF, 130^{0}C, 12h, 52%

Scheme S1: Synthesis of NDI-H and M-1

Compound 2: Mono alkylation was accomplished using a previously reported synthetic procedure.[2] On this basis, dodecyl amine (2.51 gm, 13.56 mmol) and 1-bromodecane (1.5 gm, 6.78 mmol) were mixed in methanol (25 ml) and refluxed for 24h under argon atmosphere. After that, the excess of methanol was removed under reduced pressure to afford a white semi-solid compound which was further purified by column chromatography using silica gel as stationary phase and pet-ether/ ethyl acetate (90/10) as eluent. An off white waxy material was obtained. Yield = 0.95 gm, 43%. ^{1}HNMR ($CDCl_3$, 500 MHz, TMS): δ (ppm) = 2.58 (4H, t, J = 7 Hz), 1.48 (4H, t, J = 7 Hz), 1.27-1.20 (32H, m), 0.86 (6H, t, J = 7 Hz); HRMS (ESI): m/z calc for $C_{22}H_{47}N$ $[M + H]^+$: 326.3787; found: 326.3750.

NDI-H: Naphthalene anhydride to dibromo naphthalene anhydride was prepared by following previous literature method. [3] Dibromo naphthalene anhydride was insoluble in most solvents and thus purification was not possible at this stage. The crude product (0.30 gm, 0.70 mmol) was treated with NH_4OAC (1.08 gm, 14.0 mmol) in acetic acid (15 ml) at 100˚C for 3h under argon atmosphere. After that, the reaction mixture was allowed to settle at rt and products were precipitated out in water. This product also showed poor solubility and thus did not allow purifying at this stage. The crude product (0.15 gm, 0.35 mmol) was treated with freshly prepared N-decyldodecan-1-amine (0.93 gm, 2.82mol) in DMF (8 ml) at 130˚C for 12 h. A bluish colored solution was obtained. The product was extracted with $CHCl_3$ (20 ml) and washed with acidic (1N HCl) water (40 ml). The organic layer was passed through anhydrous Na_2SO_4 and the solvent was removed under reduced pressure to obtain a bluish waxy compound. Further, the compound was purified by column chromatography using silica gel as stationary phase and 5% ethyl acetate in petroleum ether as eluent to afford a bluish waxy product. Amount obtained = 0.17 gm, Overall yield = 19 %.^{1}HNMR ($CDCl_3$, 500 MHz, TMS): δ (ppm) = 8.67 (2H, broad), 8.44 (2H, s), 3.46 (8H, t, J = 7 Hz), 1.79-0.82 (84H, m). ^{13}C NMR ($CDCl_3$) δ (ppm) =161.1, 126.0, 53.6, 32.0, 31.1, 29.7, 29.6, 29.6, 29.4, 29.4, 28.2, 27.0, 22.9, 22.8, 14.2 MALDI-TOF: m/z calc for $C_{58}H_{96}N_4O_4$ is 912.743; found 912.842.

M-1: 6-choloro-1, 3, 5 triazine-2, 6 diamine (0.20 gm, 1.37 mmol) was dissolved in dry DMF (7 ml). To this solution compound 2 (0.53 gm, 1.65 mmol), $NaHCO_3$ (0.14 gm, 1.65 mmol) were added and heated at 140˚C under argon atmosphere for 16 h. After that the reaction mixture was allowed to cool to rt. The crude reaction mixture was diluted with ethyl acetate (20 ml) and washed with brine solution (2 X 30 ml) and water (2 x 30 ml) and dried over anhydrous sodium sulphate. The excess solvent was evaporated to isolate the crude produce as a light yellow waxy material which was purified by column chromatography using silica gel as stationary phase and $CHCl_3$/ ethyl-acetate mixture as eluent. Yield: 85 %. ^{1}HNMR ($CDCl_3$, 500 MHz, TMS): δ (ppm) = 4.85 (4H, broad), 3.42 (4H, t, J= 7.5 Hz), 1.53 (4H, t, J=6 Hz), 1.35-

1.24 (32H, m), 0.87 (6H, t, J=7Hz). HRMS (ESI): m/z calc for $C_{25}H_{50}N_6$ $[M + H]^+$: 435.4194; found: 435.4171

Supramolecular polymerization model:

Nucleation-Elongation is one of the model which helps to understand supramolecular polymerization, developed by Ten Eikelder, Markvoort and Meijer [4] .This model describes the equilibrium between the monomer pool and supramolecular polymers where the cooperative growth takes place latter. It extends nucleation-elongation based equilibrium models for growth of supramolecular homopolymers to the case of two monomer and aggregate types and can be applied to symmetric supramolecular co-polymerizations, as well as to the more general case of non-symmetric supramolecular copolymerizations.

Symmetric supramolecular polymerization (dimerization)*:*

Due to the fact that supramolecular polymerization occurs via a cooperative mechanism, [4] the process can be divided in a nucleation and an elongation phase in which a nucleus size of 2 is assumed. The values T_e, ΔH°_{nucl}, ΔH° and ΔS° can be found by a non-linear least-square analysis of the experimental melting curves. The equilibrium constants associated to the nucleation and elongation phases can be calculated using equations 1 and 2

Nucleation step: $K_n = e^{\left[\frac{-(\Delta H^0 - \Delta H^0_{Nucl}) - T\Delta S^0}{RT}\right]}$ ------------- (1)

Elongation step: $K_e = e^{\left[\frac{-(\Delta H^0 - T\Delta S^0)}{RT}\right]}$ ------------ (2)

The cooperativity factor (σ) is given by: $\sigma = \frac{K_n}{K_e} = e^{\left(\frac{\Delta H^0_{Nucl}}{RT}\right)}$ -------------- (3)

Non Symmetric supramolecular polymerization developed by van der Schoot:

In the elongation regime,[5] the fraction of aggregated species (α_{agg}) can be defined by the following equation:

$$\alpha_{agg} = \alpha_{SAT}\left(1 - \exp\left[\frac{-\Delta H_e}{RT_e^2}(T - T_e)\right]\right)$$ ---------------------------- (4)

where ΔH_e is the enthalpy corresponding to the aggregation (elongation) process, T the absolute temperature, T_e the elongation temperature, R the ideal gas constant. α_{sat} is a parameter introduced to ensure that $\alpha_{agg}/\alpha_{sat}$ does not exceed unity. By using equation 4 at temperatures below the elongation temperature ($T<T_e$) it is possible to accurately fit the experimental data to the elongation regime. At the

given concentration the enthalpy release during the self-assembly of the compound in the elongation process (ΔH_e) and the elongation temperature (T_e) can be calculated.

On the other hand in the nucleation regime the fraction of aggregated species (α_{agg}) can be defined by:

$$\alpha_{agg} = K_a^{1/3} \exp\left[\left(2/3K_a^{-1/3} - 1\right)\frac{\Delta H_e}{RT_e^2}(T - T_e)\right] \text{------------------------------- (5)}$$

Where $K_a$is the dimensionless equilibrium constant of the activation step at the elongation temperature, which gives a measure of the cooperativity of the system.

In the elongation regime the number-averaged degree of polymerization, averaged over all active species $<N_n>$ can be described by the following expression:

$$< N_n >= \frac{1}{\sqrt[3]{K_a}}\sqrt{\frac{\alpha_{agg}}{\alpha_{SAT} - \alpha_{agg}}} \text{------------------------- (6)}$$

By introducing the value of K_a obtained in the nucleation and the parameters α_{sat} and α_{agg} from the elongation regimes, respectively, the number-averaged degree of polymerization $<N_n>$ can be calculated and plotted at different temperatures, according to equation 6.

The average length of the stack $<N_n>$ averaged over the nucleated species at the T_e is given by

$$\langle N_n(T_e)\rangle = \frac{1}{K_a^{1/3}} \text{ ------------------(7)}$$

The substitution of K_a in equation 7 enables the calculation of the number of aggregated molecules at the elongation temperature.

Estimation of α_{agg}:

As the values for the monomeric (ε_{mon}) and aggregate (ε_{agg}) species can be extracted from the temperature-dependent UV/Vis experiments, the fraction of aggregated species (α_{agg}) can be calculated by substituting the respective values at each temperature in the following equation:

$$\alpha_{agg} = 1 - \frac{\varepsilon - \varepsilon_{agg}}{\varepsilon_{mon} - \varepsilon_{agg}}$$

Transient absorption (TA) measurements:

TA measurements were performed with a home-built pump-probe setup. To measure in the time range of 1-4 ns with a resolution of ~100 fs, the output of a commercial titanium:sapphire amplifier (Coherent LIBRA-HE, 3.5 mJ, 1 kHz, 100 fs) was split into two beams that pumped two independent commercial

optical parametric amplifiers (Coherent OPerA Solo). One optical parametric amplifier (OPA) was used to generate the tunable excitation pulses in the visible, while the second OPA was used to generate the pump beam for white-light generation. For measurements in the spectral range between 550-1100 nm a 1300 nm seed of a few μJ was focused into a c-cut 3 mm thick sapphire window for white-light generation. The variable delay of up to 4 ns between pump and probe was introduced by a broadband retroreflector mounted on a mechanical delay stage. Mostly reflective elements were used to guide the probe beam to the sample to minimize chirp. The excitation pulse was chopped at 500 Hz, while the white-light pulses were dispersed onto a linear silicon photodiode array, which was read out at 1 kHz by home-built electronics. Adjacent diode readings corresponding to the transmission of the sample after an excitation pulse and without an excitation pulse were used to calculate $\Delta T/T$.

For measurements in the time range between 1 ns to 1 ms with a resolution of 600 ps, the excitation pulse was provided by an actively Q-switched Nd:YVO_4 laser (AOT Ltd. MOPA) at 532 nm. In this case the delay between pump and probe was controlled by an electronic delay generator (Stanford Research Systems DG535). TA measurements were performed at room temperature and samples were dissolved in THF and decane at a concentration of 0.5 mM and 1.8 mM, respectively.

Time-resolved Photoluminescence Studies:

Transient emission spectra on a picosecond timescale were taken with a Streak Camera System (Hamamatsu C4742) in fast sweep mode. The excitation wavelength of 400 nm was provided by a frequency-doubled output of a Ti: Sapphire femtosecond oscillator system (Coherent MIRA).

EPR Studies:

EPR experiment was performed in Jeol (JES FA 200) spectrophotometer. Spectra were recorded at fixed microwave frequency: 9.13 GHz, modulation width: 1 mT, microwave power: 0.998 mW, time constant: 0.03 sec. The stock solution of NDI-H (6 mM) and cobaltocene (12 mM)[6] in DCM in the glove box where we mix 125 μl of each in EPR tube. It was quickly freezed under liquid N_2. The observed peak is isotropic in nature with g = 1.99. It suggests generation of organic radical which is localized.[7]

Additional Figures:

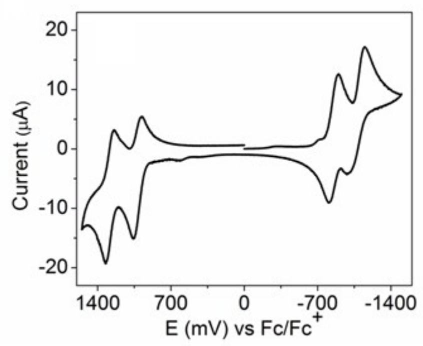

Figure S1: Cyclic voltammogram for NDI-H. Internal reference electrode: Fc/Fc^{+}; scanning rate: 100 mV/s; T = 298 K.

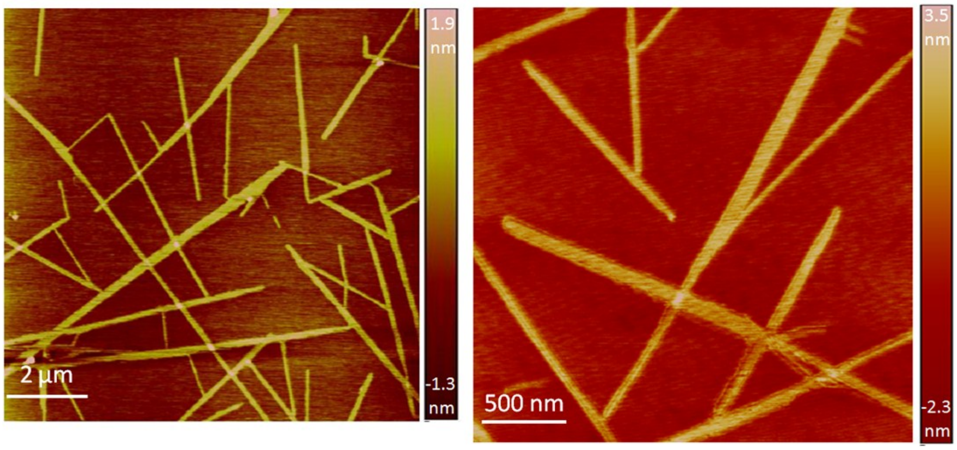

Figure S2. AFM images of diluted NDI-H gel in decane on mica surface

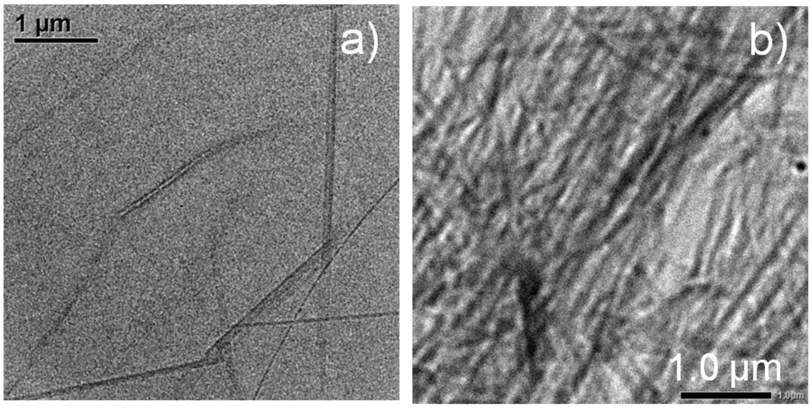

Figure S3: HRTEM images (a in 200 KV; b in 120 KV) of NDI-H in *n*-decane (*c*=0.2 mM). In 120 KV images, owing to less resolution the contrast between the wall and the interior of the tubes is less.

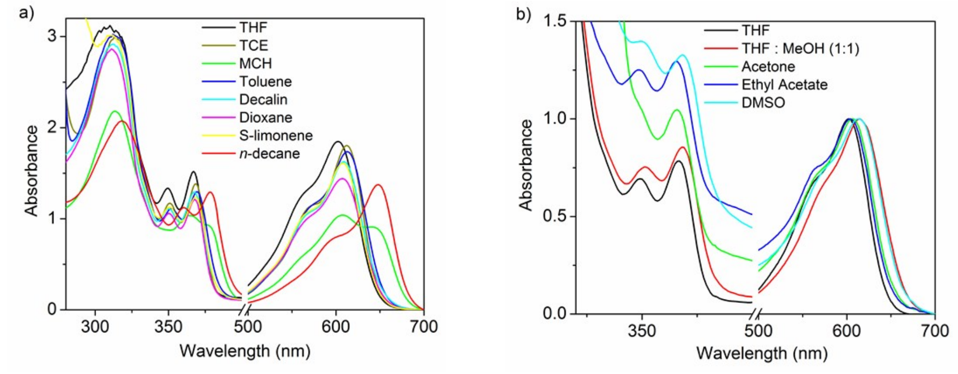

Figure S4: a) UV/Vis spectra of NDI-H (c=1.5 mM, l=0.1 cm) in non-polar solvent revealing aggregation only in MCH and decane. b) UV/Vis spectra (intensity normalized at the CT band) of NDI-H (c=0.01 mM, l= 1.0 cm) in polar solvents showing solvatochromic effects.

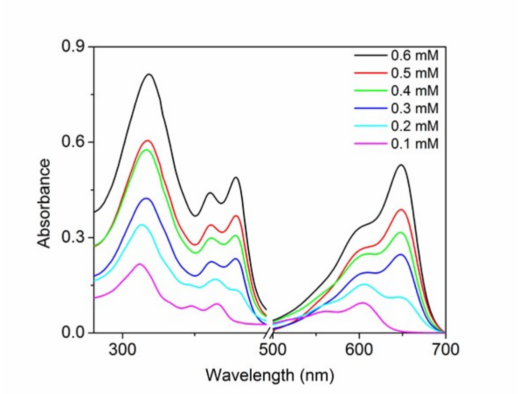

Figure S5: Concentration dependent UV/Vis study of NDI-H in *n*-decane.

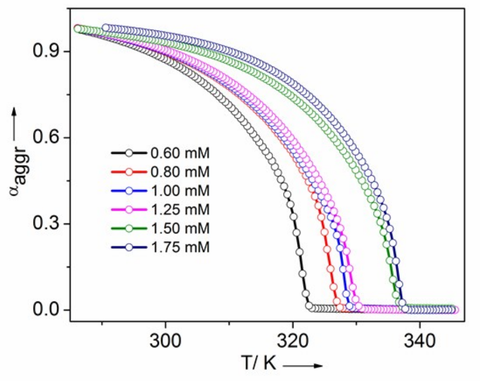

Figure S6: Cooling curves obtained by monitoring the molar absorptivity at 599 nm with temperature. In all cases, solutions of NDI-H in decane at the corresponding concentrations were heated to a monomeric state (343 K or above) and subsequently cooled down at a rate of 1.0 Kmin^{-1}to an aggregated state.

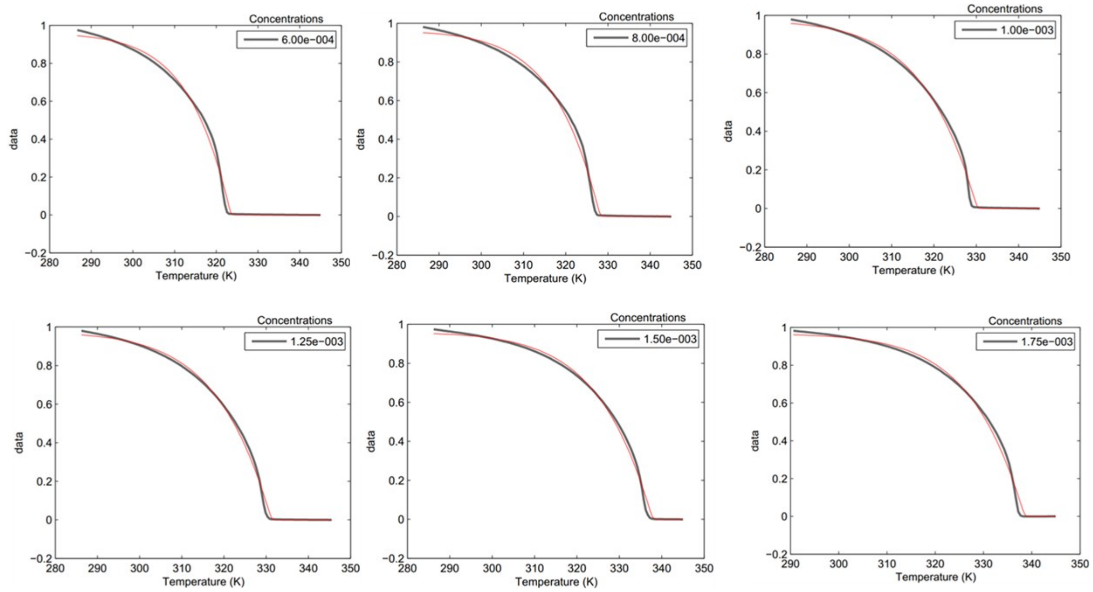

Figure S7: Cooling curves (λ = 599 nm) at different concentrations and fits to the ten Eikelder-Markvoort-Meijer cooperative model. This model assumes that the initial nucleation step is a dimerization process.

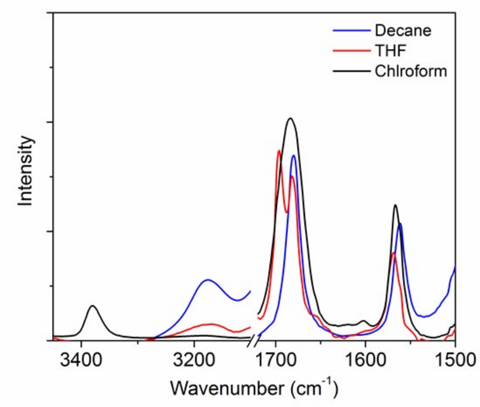

Figure S8: Solvent dependent FT-IR spectra of NDI-H (c = 1.5 mM).

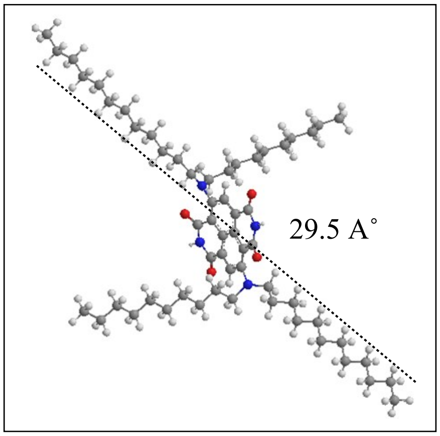

Figure S9: Energy minimized structure of NDI-H using chem. 3D ultra software.

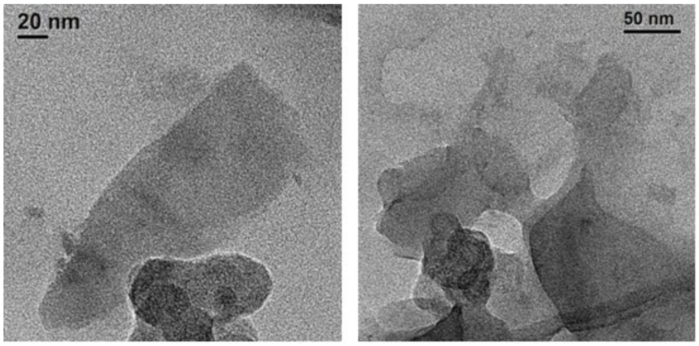

Figure S10: TEM images of NDI-H solution (10^{-6} M) in *n*-decane, dropcasted on Cu-grid.

Table S1: Results of gelation test in few organic solvents (*c*= 4mM).

THF	$CHCl_3$	Decane	TCE	Decalin	MCH	Toluene	Dioxane	S-limonene
Solution	Solution	Gel	Solution	Solution	Solution	Solution	Solution	Solution

References:

1. D. D. Perrin, W. L. F. Armarego, D. R. Perrin, *Purification of Laboratory Chemicals*, 2nd ed., Pergamon, Oxford, **1980.**

2. K. Dan and S. Ghosh, *Macromol. Rapid Commun*., 2012, **33**, 127.

3. M. Sasikumar, Y. V. Suseela and T. Govindaraju, *Asian J. Org. Chem.,* 2013, **2***,* 779.

4. H. M. M. ten Eikelder, A. J. Markvoort, T. F. A. de Greef and P. A. J. Hilbers, *J. Phys. Chem. B,* 2012, **116**, 5291; A. J. Maarkvort, H. M. M. tenEikelder, P. A. J. Hilbers, T. F. A. de Greef and E. W. Meijer, *Nature Commun.,* 2011, **2**, 509

5. P. Van der Schoot, *Supramolecular Polymers*, 2nd ed. (A. Ciferri, Taylor & Francis), London **2005**; M. M. Smulders, M. M. L. Nieuwenhuizen, T. F. A. De Greef, P. Van der Schoot, A. P. H. J. Schenning and E. W. Meijer, *Chem. Eur. J.,* 2010, **16**, 362.

6. S. T. Schneebeli, M. Frasconi, Z. Liu, Y. Wu, D. M. Gardner, N. L. Strutt, C. Cheng, R. Carmieli, M. R. Wasielewski and J. F. Stoddart, *Angew. Chem. Int. Ed*., 2013, **52**, 13100.

7. J. C. Walton, *Encyclopedia of Radicals in Chemistry, Biology and Materials* in 2012 by John Wiley & Sons, Ltd (DOI: 10.1002/9780470971253.rad001)
